# FOXF1 as an Immunohistochemical Marker of Hilar Cholangiocarcinoma or Metastatic Pancreatic Ductal Adenocarcinoma. Single Institution Experience

**DOI:** 10.3389/pore.2021.1609756

**Published:** 2021-04-20

**Authors:** Jan Hrudka, Zuzana Prouzová, Katarína Mydlíková, Kristína Jedličková, Michal Holešta, Adam Whitley, Lukáš Havlůj

**Affiliations:** ^1^ Department of Pathology, 3rd Faculty of Medicine, Charles University, University Hospital Královské Vinohrady, Prague, Czech Republic; ^2^ Clinical and Transplant Pathology Centre, Institute for Clinical and Experimental Medicine, Prague, Czech Republic; ^3^ Department of Radiodiagnostics, Charles University, 3rd Faculty of Medicine, Charles University and Královské Vinohrady University Hospital, Prague, Czech Republic; ^4^ Department of General Surgery, Charles University, 3rd Faculty of Medicine, Charles University, University Hospital Královské Vinohrady, Prague, Czech Republic

**Keywords:** intrahepatic cholangiocarcinoma, cholangiocarcinoma, Foxf1, peripheral, hilar, extrahepatic

## Abstract

Cholangiocarcinoma (CCA) is a liver malignancy associated with a poor prognosis. Its main subtypes are peripheral/intrahepatic and hilar/extrahepatic CCA. Several molecular, morphological and clinical similarities between hilar/extrahepatic CCA and pancreatic ductal adenocarcinoma (PDAC) have been described. FOXF1 is a transcription factor which has been described to have prognostic significance in various tumors and it is involved in the development of bile ducts. The aim of this study is to determine occurrence of nuclear expression of FOXF1 in both subtypes of CCA and metastatic PDAC and assess its potential usefulness as a diagnostic marker. Secondary aims were to investigate the use of C-reactive protein (CRP) immunohistochemistry for diagnosing intrahepatic peripheral CCA and the significance of histological features in CCA subtypes. 32 archive specimens of CCA, combined hepatocellular carcinoma-CCA (HCC-CCA) and liver metastasis of PDAC were stained by FOXF1 and CRP immunohistochemistry and evaluated to determine histological pattern. The CCAs were classified radiologically into peripheral/intrahepatic and hilar subtype. Using Fisher exact test, we identified nuclear FOXF1 as a fairly specific (87%) but insensitive (65%) marker of hilar and extrahepatic CCA and metastatic PDAC (*p* = 0.005). CRP immunohistochemistry was characterized by a high sensitivity and specificity, of 79% and 88%, respectively (*p* = 0.001). We did not identify any histomorphological features associated with either types of CCA or metastatic PDAC. As a conclusion of novel finding, FOXF1 immunohistochemistry may be regarded as a specific but insensitive marker of hilar/extrahepatic CCA and metastatic PDAC and it may help distinguish them from peripheral CCA.

## Introduction

Cholangiocarcinoma (CCA) is a relatively rare malignancy with a generally poor prognosis, often attributed to advanced stage at the time of diagnosis. The only potential cure is radical surgery. According to the World Health Organization (WHO) classification of gastrointestinal tumors, cholangiocarcinoma is regarded as a primary tumor arising from any part of the biliary tree including the intrahepatic bile ducts, gallbladder and extrahepatic bile ducts [1–3]. The left and the right hepatic bile ducts and their first to third branches are called hilar or perihilar bile ducts. These are followed proximally by the intrahepatic bile ducts. Intrahepatic CCA is a liver tumor that may arise in any part of an intrahepatic biliary tree. Intrahepatic carcinoma may be further subdivided into a peripheral subtype, which arises from the small intrahepatic bile ducts, and a hilar subtype, which involves the left or right bile duct or their junction. Combined hepatocelullar-cholangiocarcinoma (HCC-CCA) is a rare variant that closely resembles peripheral CCA [4].

Hilar CCA shares morphological, molecular and prognostic features with extrahepatic CCA [5] and pancreatic ductal adenocarcinoma (PDAC) [6, 7]. Peripheral intrahepatic CCA, on the other hand, has morphological, molecular and clinical similarities to combined HCC-CCA [4, 7]. Hilar and extrahepatic CCA and PDAC usually affect older people, are frequently recognized in advanced stages when they are unresectable and have a dismal prognosis. Histologically, these tumors display tubular, cribriform and tubulo-papillary arranged cylindrical epithelium. They express the immunohistochemical markers S100P and AGR2 [7, 8]. Peripheral CCA occurs in the peripheral parts of the liver as a large mass and microscopically consists of cuboid epithelium with anastomosing or trabecular architecture. Reliable makers for detecting of peripheral CCA include CRP immunohistochemistry [9] and *in situ* hybridization of albumin messenger RNA [10].

Distinction between peripheral CCA, hilar CCA and metastatic PDAC is of great clinical relevance because they are treated differently and have different prognoses. In many cases, both CCA subtypes may be distinguished radiologically by contrast-enhanced computed tomography (CE-CT) and magnetic resonance (MRI). Generally, CT and MRI have high sensitivity in detecting mass forming liver and pancreatic tumors but suffer from relatively low specificity in regard to predicting the histological type of the tumor. Differentiating between hilar and peripheral CCA can also be difficult, especially for large tumors [11]. Hilar and extrahepatic CCA and PDAC have a worse prognosis than intrahepatic peripheral CCA [6]. Resectability of CCA varies among institutions between 10 and 75% [12]. The extent and type of surgery differs substantially depending on the anatomical subtypes of CCA. Patients with metastatic PDAC do not benefit from surgical resection.

A histological or immunohistochemical marker of both peripheral and hilar type of CCA or metastatic PDAC may lead to accurate diagnosis in the case of unclear radiological finding. The forkhead box f1 (FOXF1, previously known as HFH-8 or Freac-1) is a homeobox gene that encodes a transcription factor expressed in the extraembryonic mesoderm, allantois, splanchnic mesoderm, and septum transversum mesenchyme [13]. It is involved in mesenchymal-epithelial signaling required for development of structures that arise from the foregut endoderm, such as the lung, gallbladder, and pancreas. FOXF1 haploinsufficiency has been described to cause malformations of pulmonary vessels and the colon in mice [14, 15]. Several recent works have linked FOXF1 deletions to lethal alveolar capillary dysplasia with misalignment of pulmonary veins (ACDMPV) in human infants [16–20]. Kalinichenko et al. detected expression of FOXF1 in the septum transversum and gall bladder in mice. In their study, mice with heterozygous expression of FOXF1 had severe structural abnormalities of the gallbladder and extrahepatic bile ducts involving both mesenchymal and epithelial parts of the organs [21]. FOXF1 expression has also been associated with high grade and advanced stage colorectal cancer [22].

All facts mentioned above concerning importance of FOXF1 in extrahepatic bile ducts and pancreas development lead us to the idea to test expression of FOXF1 in CCAs and metastatic pancreatic carcinomas by immunohistochemistry. The aim of this study is to discover if there is any difference in FOXF1 expression in the peripheral subtype of CCA vs. hilar and extrahepatic CCA and liver metastasis of PDAC. We hypothesize that FOXF1 may be expressed in the latter group of tumors due to its involvement embryonic development of distal bile ducts and pancreatic ducts.

## Material and Methods

### Patient Selection

The medical records of the department of pathology of the University Hospital Královské Vinohrady were reviewed to find all biopsies and resected specimen of CCA, combined HCC-CCA and liver metastasis of PDAC from years 2010–2020. A total of 36 cases were identified. These consisted of 20 male and 16 female subjects, with a mean age of 65 years (median 66.5, range 38–80, standard deviation 10.9). The samples consisted of 22 needle liver biopsies, 12 excisions or partial liver resection specimens, one cholecystectomy specimen and 1 excision from a CCA metastasis in the greater omentum (Supplementary Table S1). The project was approved by institution`s ethical committee (approval number 09/0/2020).

### Grouping of Cases

**FIGURE 1:**
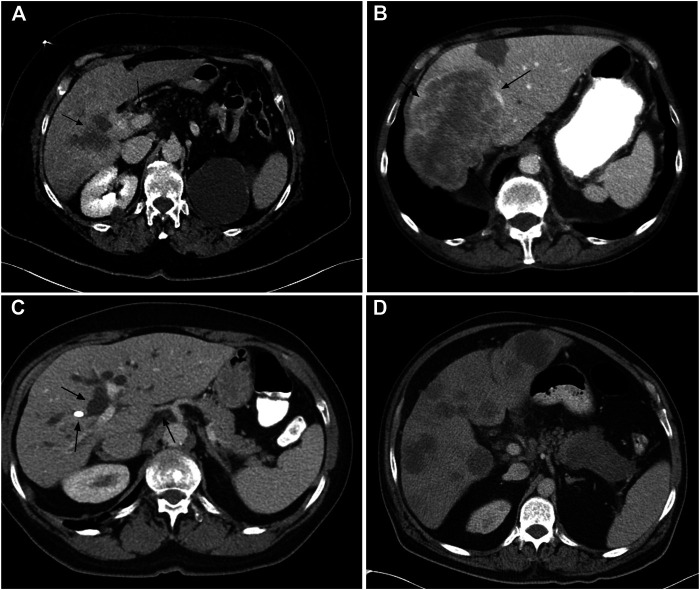
CT scans from the study showing: Mass forming hilar CCA with infiltration along portal vein **(A**, arrows toward the tumor mass**)**; Large mass forming peripheral CCA **(B**, arrows toward the tumor mass**)**; Hilar CCA with bile duct dilatation, stent and infiltration along hepatic artery **(C**, arrows showing the bile duct dilatation**)**; Pancreatic tail tumor with multiple liver metastases **(D)**.

In all cases, computed tomography (CT) and magnetic resonance tomography (MRT) scans were evaluated by an experienced radiologist (MH) with the aim to distinguish between peripheral CCA, hilar CCA, extrahepatic CCA and PDAC liver metastases (Figure 1). To assess the relative frequency of FOXF1 and CRP expression and the histomorphological appearance in particular radiological diagnoses we divided the specimens into two groups. Group 1 consisted of the peripheral CCAs and combined HCC-CCA. 15 cases were allocated to this group 1. Group 2 consisted of hilar and extrahepatic (including gallbladder) CCAs and metastatic PDAC. Group 2 consisted of a total of 17 cases, seven of which were metastatic PDAC. In four cases we were not able to distinguish between hilar and extrahepatic tumors based on radiological findings—these were excluded from further evaluation.

### Histology and Immunohistochemistry

**FIGURE 2:**
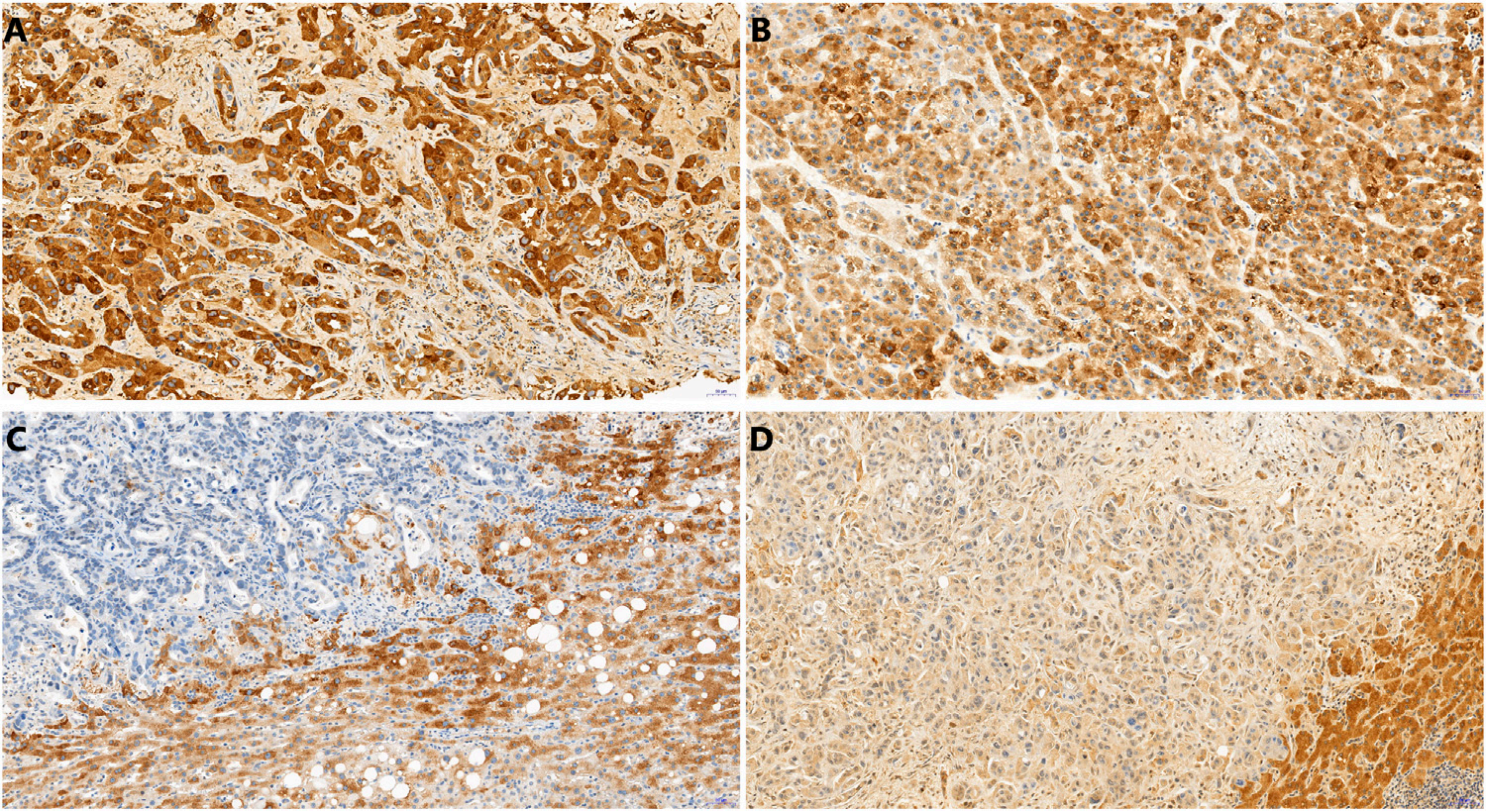
CRP immunohistochemistry showing positive peripheral intrahepatic CCA **(A)**, positive combined HCC-CCA **(B)**, negative hilar CCA **(C)**, negative metastasis of PDAC **(D)**. In **C** and **D**, note the cytoplasmic positivity in non-neoplastic liver tissue. 20x.

**FIGURE 3:**
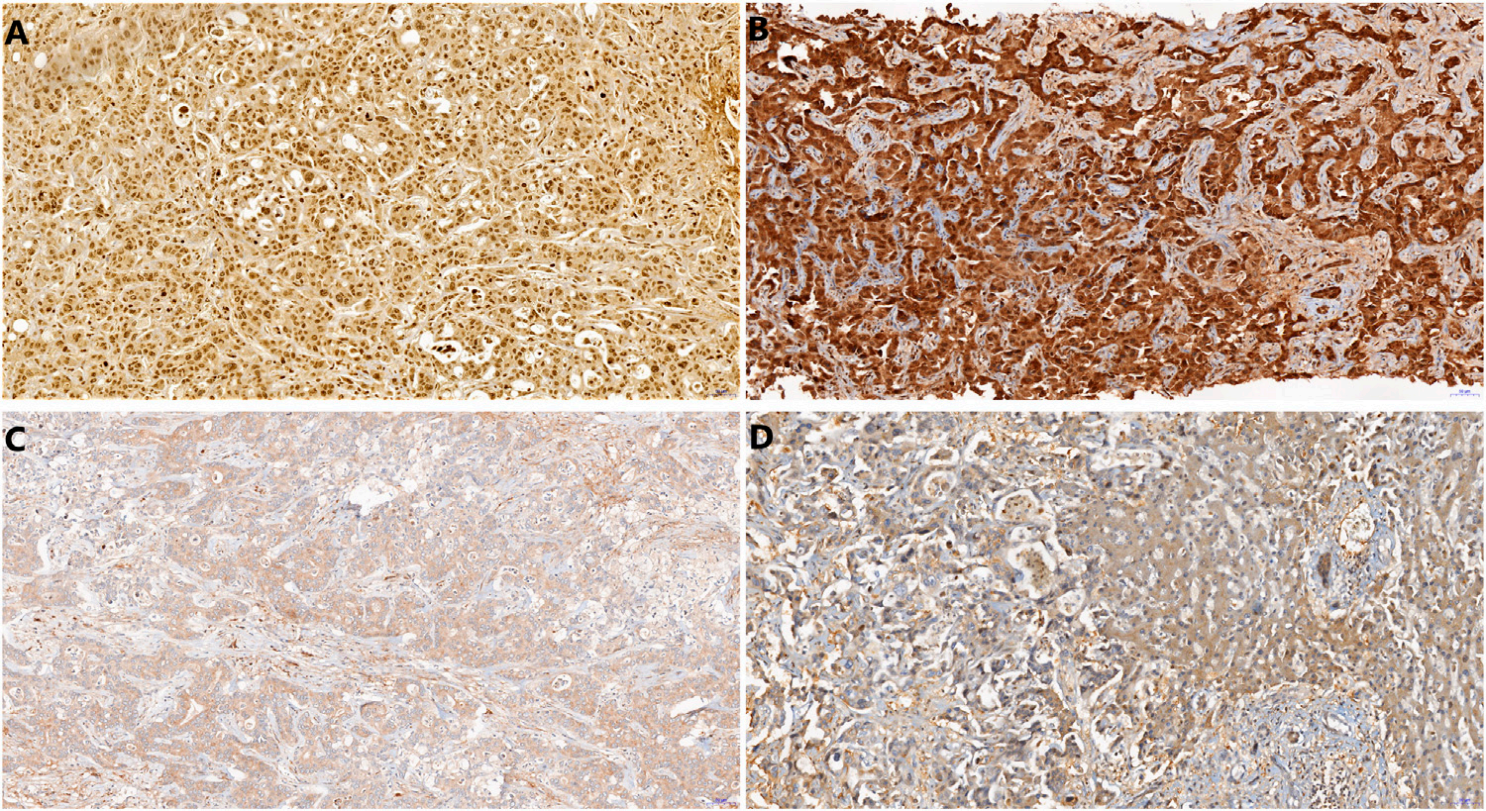
FOXF1 immunohistochemistry showing nuclear positivity in PDAC metastatic to liver **(A)**, both nuclear and cytoplasmic positivity in hilar CCA **(B)**, negativity in peripheral intrahepatic CCA **(C)** and negativity in combined HCC-CCA **(D)**. In the right part of **D**, note the negativity in non-neoplastic liver tissue. 20x.

**FIGURE 4:**
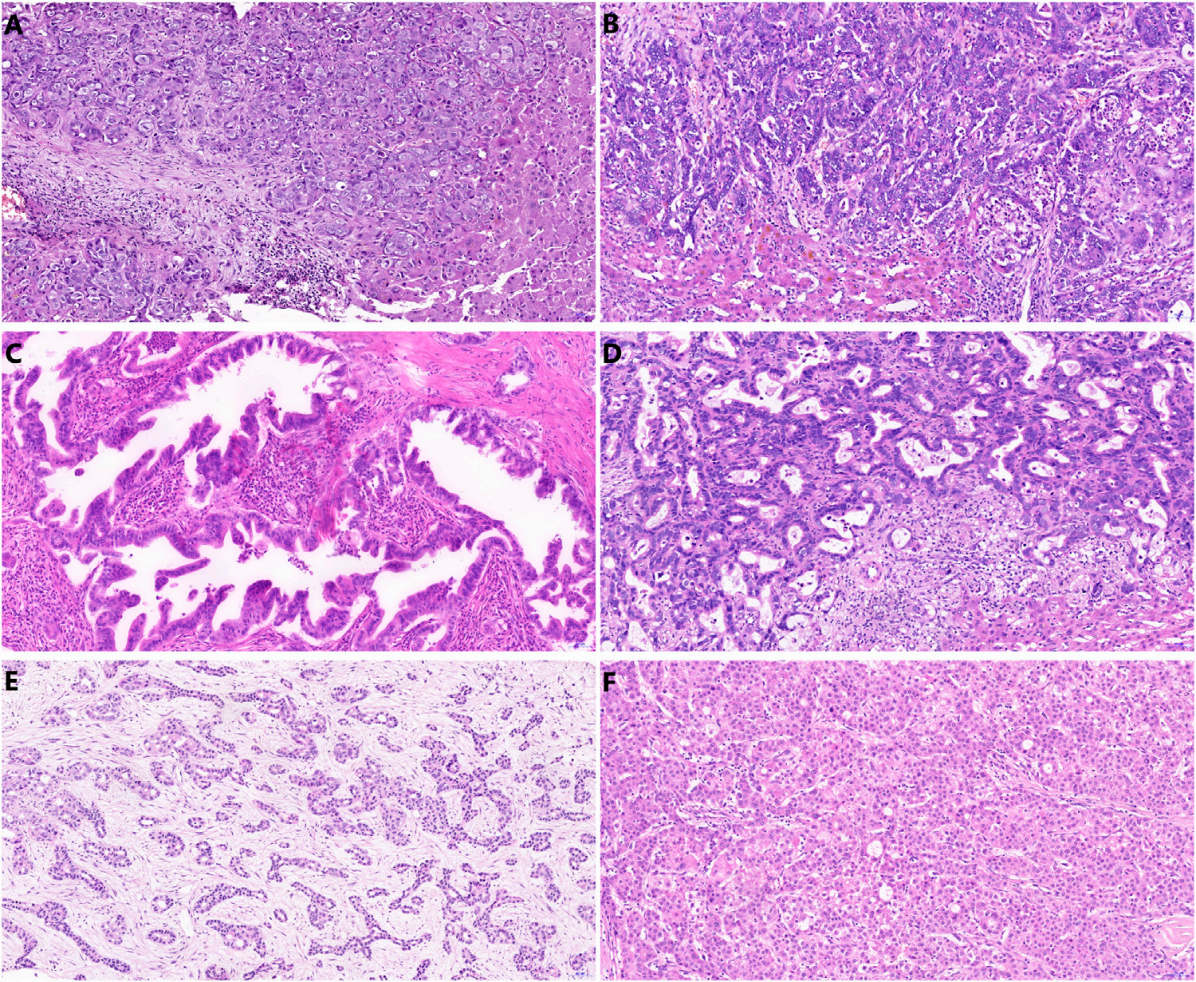
Hematoxylin eosin slides showing variable histomorphological pattern: solid trabecular **(A)** and anastomosing pattern in peripheral CCA **(B)**, tubulopapillary pattern **(C)** and anastomosing-tubular pattern in hilar CCA **(D)**, arguable pattern finally evaluated as anastomosing-trabecular in peripheral CCA **(E)** and trabecular pattern in combined HCC-CCA **(F)**.

Archive formalin fixed paraffin embedded biopsy material was used for morphological and immunohistochemical analysis. Immunohistochemical detection of CRP (polyclonal antibody, ABCAM, ab 31,156, 1:200) was performed on 4-μm thick sections of paraffin-embedded tissues using the Ventana Benchmark Ultra automated stainer (Tucson, AZ, United States) with OptiView DAB IHC Detection Kit. Immunohistochemical detection of FOXF1 (polyclonal, Abcam, 1:50) was performed on 4-μm thick sections of paraffin-embedded tissues using the Ventana Benchmark Ultra automated stainer with Ultraview Detection System (Ventana Medical Systems). The slides were counterstained with hematoxylin. Stained slides were dehydrated and covered in a xylene-based mounting medium. Morphological studies were performed using routine hematoxylin-eosin stained slides. The microscopic analysis was performed by two experienced routine histopathologists (JH and ZP). Unclear cases were settled by mutual consideration. All analyses were performed without knowledge of the clinical setting and radiological findings. For CRP detection cytoplasmic staining was considered positive (Figure 2). We distinguished nuclear and cytoplasmic positivity for FOXF1 immunohistochemistry (Figure 3). Concurrent cytoplasmic and nuclear positivity was recorded as nuclear. When evaluating histomorphological appearance, it was noted whether the samples had non-anastomosing and anastomosing patterns. The morphological appearance was further sub-grouped into glandular (tubular, microtubular and cribriform) or solid (trabecular) subtypes, regardless as to whether the tumor had an anastomosing or non-anastomosing pattern (Figure 4).

### Statistics

We evaluated the relations of FOXF1 expression, CRP expression, histological pattern (tubular vs. trabecular-anastomosing) in relation to radiological groups. All cases are listed in Supplementary Table S1, the cases are ordered according to date of biopsy or surgery (not published). All cases used in the study with group assignment are listed in Table 1. To evaluate the mean age of patients in both radiological groups, we used Student *t*-test. To evaluate the association of FOXF1, CRP and histomorphology with the radiological group, we used Fisher exact test. We considered *p* values < 0.05 statistically significant.

**TABLE 1:** list of all examined cases and analyzed variables.

Group	Gender	Age	Type of specimen	Imaging method	Foxf1	CRP	Histology
1	M	38	Needle biopsy	Peripheral CCA	Cytoplasmic only	Positive	Ductal papillary
1	M	46	Needle biopsy	Peripheral CCA	Negative	Positive	Trabecular anastomosing
1	F	55	Resection	Peripheral CCA	Negative	Positive	Trabecular anastomosing
1	M	64	Resection	Peripheral CCA	Negative	Positive	Cribriform anastomosing
1	M	66	Needle biopsy	Peripheral CCA	Negative	Positive	Trabecular anastomosing
1	M	67	Needle biopsy	Peripheral CCA	Negative	Positive	Tubular anastomosing
1	F	68	Needle biopsy	Peripheral CCA	Negative	Positive	Trabecular anastomosing
1	F	68	Needle biopsy	Peripheral CCA	Cytoplasmic only	Positive	Trabecular anastomosing
1	M	69	Resection	Peripheral CCA	Negative	Negative	Cribriform anastomosing
1	M	73	Resection	Peripheral CCA	Negative	Negative	Tubular anastomosing
1	M	78	Resection	Combined HCC-CCA	Negative	Positive	Trabecular anastomosing
1	M	78	Needle biopsy	Peripheral CCA	Nuclear	Material lost	Tubular anastomosing
1	F	78	Needle biopsy	Peripheral CCA	Nuclear	Positive	Tubular anastomosing
1	F	79	Resection	Peripheral CCA	Negative	Positive	Tubular
1	M	80	Needle biopsy	Peripheral CCA	Cytoplasmic only	Positive	Tubular anastomosing
2	F	39	Resection	Hilar CCA	Negative	Negative	Cribriform anastomosing
2	M	50	Needle biopsy	Hilar CCA	Nuclear	Positive	Trabecular anastomosing
2	M	52	Needle biopsy	PDAC metastasis	Nuclear	Negative	Trabecular anastomosing
2	F	58	Resection	Hilar CCA	Cytoplasmic only	Negative	Tubular
2	M	58	Resection	PDAC metastasis	Nuclear	Negative	Trabecular anastomosing
2	F	60	Needle biopsy	PDAC metastasis	Nuclear	Negative	Cribriform anastomosing
2	M	63	Needle biopsy	PDAC metastasis	Nuclear	Negative	Trabecular anastomosing
2	M	64	Resection	Hilar CCA	Negative	Negative	Trabecular anastomosing
2	M	64	Metastasis omentum	Hilar CCA	Nuclear	Negative	Tubular
2	F	65	Needle biopsy	Hilar CCA	Nuclear	Negative	Tubular anastomosing
2	F	66	Resection	Hilar CCA	Negative	Negative	Trabecular anastomosing
2	M	67	Needle biopsy	PDAC metastasis	Nuclear	Negative	Tubular anastomosing
2	F	69	Gall bladder	Gall bladder CCA	Cytoplasmic only	Negative	Tubular
2	F	75	Resection	Hilar CCA	Negative	Negative	Tubular
2	F	75	Needle biopsy	Hilar CCA	Nuclear	Positive	Trabecular anastomosing
2	M	77	Needle biopsy	PDAC metastasis	Nuclear	Negative	Cribriform anastomosing
2	M	80	Needle biopsy	PDAC metastasis	Nuclear	Negative	Cribriform anastomosing

CRP, C-reactive protein; CCA, cholangiocarcinoma; HCC, hepatocellular carcinoma; PDAC, pancreatic ductal adenocarcinoma.

## Results

Concerning age of patients, the mean was 67.1 years in Group 1 and 63.7 years in Group 2, the difference is not significant (*p* = 0.3956).

### FOXF1

Nuclear expression of FOXF1 (Figure 3) was more frequently identified in Group 1 (*p* = 0.005). Only 2 cases of nuclear FOXF1 were identified in Group 1 and both were peripheral CCAs, whereas 11 were identified in Group 2 (Table 2). The sensitivity and specificity of nuclear FOXF1 staining as a marker of hilar/extrahepatic CCA and PDAC were 64.7% and 86.7%, respectively. When taking all cases of nuclear and cytoplasmic FOXF1 staining together there was slighter but still significant difference between the two groups (*p* = 0.03); there were 5 cases in Group 1 (all were peripheral CCAs) with positive FOXF1 and 13 cases in Group 2 (Table 2). The sensitivity and specificity of both nuclear and cytoplasmic FOXF1 staining as a marker of hilar/extrahepatic CCA and PDAC were 76.5% and 66.7%, respectively. The nuclei of non-neoplastic liver cells were FOXF1-negative.

**TABLE 2:** Fisher exact test counts evaluating FOXF1 expression in Group 1 (intrahepatic/peripheral + combined HCC/CCA) vs. Group 2 (hilar/extrahepatic CCA + metastatic PDAC), comparing distrinbution of nuclear staining and both cytoplasmic and nuclear staining.

	FOXF1+ (nuclear)	FOXF1− (nuclear)	FOXF1+ (nuclear and cytoplasmic)	FOXF1− (nuclear and cytoplasmic)
Group 1	2	13	5	10
Group 2	11	6	13	4
	*p* = 0.005		*p* = 0.03	

### CRP

There was a significant difference in CRP staining (Figure 2) between the two groups (*p* = 0.001). There were 12 cases in Group 1 that displayed with cytoplasmic positivity for CRP. In one case the material from needle biopsy was lost during CRP immunohistochemistry processing. The other 2 cases from Group 1 were negative. 2 cases in Group 2 stained positive for CRP (Table 3). The sensitivity and specificity of CRP as a marker of intrahepatic peripheral CCA and combined HCC-CCA were 78.6% and 88.2%, respectively. As a control, the cytoplasmic CRP positivity was noted in the non-neoplastic hepatocytes.

**TABLE 3:** Fisher exact test counts evaluating CRP expression in Group 1 (intrahepatic/peripheral + combined HCC/CCA) vs. Group 2 (hilar/extrahepatic CCA + metastatic PDAC), cytoplasmic staining is considered positive.

	CRP+	CRP−
Group 1	12	2
Group 2	2	15
*p* = 0.001		

### Histomorphology

In Group 1, there were 8 cases with anastomosing or cribriform architecture (including combined HCC-CCA) and 7 cases with tubular or microtubular structure (Figures 4A,B,E,F). In Group 2, there were 13 cases showing anastomosing or cribriform pattern and 4 cases with a tubular arrangement (Figures 4C,D). There was no significant difference in the microscopic architecture between the two groups (*p* = 0.266). In terms of glandular-tubular-cribriform vs. solid-trabecular subtypes, there were 6 cases in Group 1 with trabecular pattern and 9 cases with glandular arrangement. In the Group 2 there were 7 cases showing trabecular histology and 10 cases with apparent glandular structures. There was no significant difference in these histological subtypes between the two groups (*p* = 1).

## Discussion

Morphological, histochemical and clinical similarities between hilar and extrahepatic CCA and PDAC have been described in recent studies [7]. Macroscopically, these tumors are frequently seen infiltrating the peribiliary soft tissue and growing along the bile ducts, whereas intrahepatic peripheral CCA more frequently manifests as a mass lesion [1]. Microscopically, hilar and extrahepatic CCAs have cylindrical epithelium and tubular architecture in contrast to the cuboidal epithelium and trabecular arrangements observed in intrahepatic peripheral CCAs. On the other hand, intrahepatic peripheral CCA and combined HCC-CCA tend to show anastomosing and trabecular histology and expression of hepatocellular markers such as CRP [9].

The differences in macroscopic, microscopic, histological, molecular and clinical properties of both CCA subtypes, combined HCC-CCA and PDAC may be explained by the different origins of these tumors. Hilar and extrahepatic CCA and PDAC are believed to arise from multipotent progenitor cells located in the peribiliary glands around the bile and pancreatic ducts [7]. These cells are of endodermal origin and are able to differentiate into hepatocytes, cholangiocytes and pancreatic cells [23]. Intrahepatic small bile ducts arise from hepatic progenitor cells via ductal plates at the terminal portal tracts [24]. The rare combined HCC-CCA may be proof of the continuous nature of liver tumor differentiation in the sense of hepatocellular-cholangiocellular phenotype. The biliary tract and pancreas are anatomically closely related. Embryologically, the extrahepatic bile ducts and the ventral pancreas arise from the diverticulum of the posterior ventral foregut almost at the same time and several transcription factors, such as Pdx1, Hes1, SOX9, and SOX17, are sequentially involved in the biliary and pancreatic differentiation [23]. There are studies documenting developmental defects of the pancreato-biliary system in SOX17-and Hes1-deficient mice [25, 26]. FOXF1 is a transcription factor involved in foregut and extrahepatic bile duct development. It is expressed mainly in fibroblasts, smooth muscle cells and endothelium [27, 28]. Several studies have described developmental defects of the extrahepatic bile ducts and gallbladder in FOXF1-deficient mice [21]. Moreover, FOXF1 has been described to play a significant role in cancerogenesis and cancer promoting molecular processes in colorectal cancer [22, 29, 30]. Expression of FOXF1 in lung cancer fibroblast promotes the cancer invasion and spread [31]. In HCC and breast cancer FOXF1 has been described as a tumor suppressor [32, 33]. On the other hand, FOXF1 has also been shown to be involved in tumor progression. In breast and lung cancer it has been shown to induce epithelial-mesenchymal transition, a crucial step in metastasis [27, 34]. All these findings suggest that the role of FOXF1 in tumorigenesis is extremely complex and tissue-specific. The association of FOXF1-deficiency with biliary tract malformations described in mice [21] and its importance in various carcinomas led us to use FOXF1 antibody as a potential immunohistochemical marker of CCA and metastatic PDAC. Moreover, we evaluated the histological pattern and CRP expression as a marker of intrahepatic peripheral CCA.

The results of our study display unreliability of pure histomorphology as there was no significant association in trabecular-anastomosing or tubular arrangement with the CCA subtype or metastatic PDAC. This finding does not corroborate findings by Gandou et al., who observed that columnar-tubular pattern was more frequently associated with perihilar CCA and PDAC and cuboidal-trabecular pattern was more frequently associated with intrahepatic peripheral CCA and combined HCC/CCA [6].

Although CRP immunohistochemistry was not the main aim of our study, we confirmed the association of CRP expression with intrahepatic peripheral CCA, which has been well described by Yeh et al. In their study, the sensitivity and specificity of CRP expression in the diagnosis of iCCA were 75.7% and 91.1% when using tissue microarrays and 93.3% and 88.2% when using whole tissue sections, respectively [9]. Our cohort displayed surprisingly similar sensitivity and specificity of 78.6% and 88.2%, respectively. Additionally to the corroboration of previous studies, this finding may signify accuracy of radiological diagnosis in our study.

To the best of our knowledge, this is the first study investigating FOXF1 as an immunohistochemical marker of CCA or metastatic PDAC. Our result suggests that FOXF1 is more frequently expressed in hilar and extrahepatic CCAs and metastatic PDACs in comparison to intrahepatic peripheral CCA and combined HCC/CCA. As an nuclear immunohistological marker, FOXF1 shows a fair specificity of 86.7% (only a few nuclear FOXF1 positive CCAs were peripheral) but low sensitivity of 64.7% (two thirds of hilar/extrahepatic CCAs and metastatic PDACs were FOXF1 negative). If we regard only nuclear positivity, FOXF1 may be labeled as a specific but insensitive marker of hilar and extrahepatic CCA or metastatic PDAC. Concerning cytoplasmic positivity of FOXF1, the cytoplasmic vs. nuclear localization of this transcription factor has been described to correlate with progression of colorectal cancer [22]. In the case of CCAs and PDACs, the significance of intracellular compartmentalization of FOXF1 remains unclear. However, our study showed lower statistical significance (*p* = 0.03) of cytoplasmic and nuclear FOXF1 to discern between CCA subtypes and metastatic PDAC in comparison with nuclear positivity only. The cytoplasmic and nuclear FOXF1 staining as a method to uncover hilar/extrahepatic CCA and metastatic PDAC showed higher sensitivity (76.5%) but poor specificity (66.7%) in comparison with solely nuclear staining. Like in many transcription factors detectable by immunohistochemistry used in the human pathology (i.e. CDX2, PAX2, PAX8, BOB1, SOX10, SOX11, SALL4, SATB2 etc.), we regard only nuclear FOXF1 positivity to have a diagnostic value; whereas the significance of cytoplasmic FOXF1 localization needs to be further clarified.

A surprising finding was the unreliability of histomorphological pattern in distinguishing the subtypes of CCA. From all the methods tested in the study, radiological finding, together with immunohistochemical CRP positivity may serve as hallmark features when making the diagnosis of intrahepatic peripheral CCA. In line with recent publications, there is no histopathological method to distinguish between hilar or extrahepatic CCA and metastatic PDAC. Our study is limited by its relatively small cohort size. However, there is a space for further research to identify diagnostic histopathological tests for both subtypes of CCA.

## Data Availability

The datasets presented in this study can be found in online repositories. The names of the repository/repositories and accession number(s) can be found in the article/Supplementary Material.
